# Qualitative evaluation to improve participant engagement and retention in remote COVID-19 treatment trials

**DOI:** 10.1186/s12982-025-00970-3

**Published:** 2025-09-24

**Authors:** Amaya Perez-Brumer, Rebecca Balasa, Aarti S. Doshi, Andrea Bowra, Julien Brisson, Morgan M. Philbin, Thuy Doan, Catherine E. Oldenburg

**Affiliations:** 1 Dalla School of Public Health, University of Toronto, Toronto, ON M5T 3M7 Canada; 2 Division of Vulnerable Populations, Department of Medicine, University of California, San Francisco, CA 94158 USA; 3 Francis I. Proctor Foundation, University of California, San Francisco, CA 94158 USA; 4 Department of Ophthalmology, University of California, San Francisco, CA 94158 USA; 5 Department of Epidemiology & Biostatistics, University of California, San Francisco, CA 94158 USA; 6 Institute for Global Health Sciences, University of California, San Francisco, CA 94158 USA

**Keywords:** COVID-19 research, Clinical trial, Qualitative evaluation, Recruitment, Retention, Motivations

## Abstract

Clinical trials, although recognized as the gold standard for scientific research, encounter various challenges with participant recruitment and retention. Yet, given the surge in global research endeavors during the COVID-19 pandemic, mass recruitment and retention were needed to ensure study power. While participant engagement in clinical research has been investigated, there is limited literature underscoring motivations for participation in COVID-19 clinical trials conducted remotely. To address this gap, we conducted a longitudinal, qualitative evaluation study of participant engagement and retention in a remote, COVID-19 clinical trial. In-depth, semi-structured interviews were conducted between December 2020-March 2021 (timepoint 1; *n* = 19) and January–February 2022 (timepoint 2; *n* = 11) to gain insights from participants regarding their experiences, motivations, and challenges with clinical trial engagement during the COVID-19 pandemic. Findings identified several factors described to increase participant engagement, including perceived contribution to the greater good, protecting oneself and others, and gaining improved access to healthcare. Further, key themes underscoring the enhancement of retention, such as perceived trial-related challenges and recommendations for future studies, were also identified. Findings underscore the utility of embedding qualitative methods within clinical trials, and how they contribute to a broader understanding of factors influencing participation in COVID-19 trials while providing valuable insights to enhancing recruitment and retention strategies. Consequently, this study provides essential knowledge aimed at optimizing participant engagement and retention in clinical trials, thereby advancing our comprehension of and response to research mobilization during unprecedented events.

## Introduction

Clinical trials are recognized as the gold standard for assessing the safety and effectiveness of both new medical interventions as well as existing medical interventions for novel health conditions. Despite considerable efforts and resources employed towards recruitment strategies, the methodological challenges of recruiting clinical trial participants are often understated [1]. Findings have shown that over two-thirds of clinical trials conducted in developed countries have fallen short of recruiting expected numbers of clinical trial participants [2], and over 25% of clinical trials undergo early termination due to low numbers in participant recruitment [3]. Following the exogenous shock of the COVID-19 pandemic, rapid mobilization of the scientific community led to a worldwide surge in COVID-19 clinical trials [4–6]. To gain a better understanding of the disease and develop effective measures to address its impacts, additional strategies were employed to enhance rapid recruitment and participation worldwide to ensure study power [7].

According to the World Health Organization’s [8] International Clinical Trials Registry Platform, there are currently over 20,000 active or completed COVID-19-related studies worldwide as of June 2023. These clinical trials have explored various aspects of the disease, such as its transmission, short and long-term effects, diagnostic techniques, as well as established and innovative treatment and prevention methods. COVID-19 research has played a crucial role in equipping us with essential tools for mitigating negative outcomes, including rapid testing methods such as rapid antigen tests (RAT), polymerase chain reaction (PCR) tests, and vaccines, among others. Crucially, it is imperative to underscore the pivotal role of participants who voluntarily partake in COVID-19 clinical trials and the need to actively learn from their experience to improve participation in medical research more broadly.

COVID-19 research studies have employed various recruitment methods, yielding different outcomes of success. Three prominent trials—*RECOVERY*, *SOLIDARITY*, and *ACTT*—achieved relatively high success rate in recruiting COVID-19 patients admitted to hospitals [9–11]. However, this strategy only captured clinical samples who tested positive for COVID-19 and sought medical care at the designated study sites. To recruit community- and population-based samples, trials began employing traditional participant recruitment methods, such as distributing flyers, sending letters, contacting employers, and leveraging social media advertising [12, 13]. Moreover, novel participant recruitment approaches have been implemented to overcome challenges specific to COVID-19 trials. One example, a “Data Mart”, involves the storage of aggregated data collected from a centralized electronic health record system (EHR), permitted via a health information exchange (HIE) system and patient’s consent-to-contact permissions, to rapidly screen eligible participants from a large pool of candidates and conduct separate research studies in inpatient and/or outpatient settings [14]. Helmer et al. developed a COVID-19 Recruitment Data Mart at Vanderbilt University Medical Center to collect and store outpatient EHR in their Research Electronic Data Capture (REDCap) system to facilitate participant recruitment across four COVID-19 studies onboarded between July 2021-March 2022 [15]. Considering the overlapping pools of eligible participants for COVID-19 studies and the urgency produced by the COVID-19 pandemic, these strategies aimed to regulate and expedite participant recruitment for multiple trials [14–16].

Research conducted prior to COVID-19 has extensively examined barriers and facilitators to clinical trial participation, especially in realm of cancer and HIV research [17–21], highlighting key factors that influence decisions on whether or not to participate in research [22–26]. A systematic review focusing on barriers to cancer clinical trial participation identified logistical reasons for non-participation, such as eligibility criteria and geographical limitations [21]. Literature has also emphasized trustworthiness surrounding health systems, medicine, and clinical trials as a facilitator for research participation, necessitating transparency between investigators/research personnel and participants [27, 28]. Another systematic review of qualitative studies that examined perspectives of and motivations for clinical trial participation underscored “building confidence” [29] as a key theme for facilitating recruitment. Encompassed within this theme were examples pertaining to the trustworthiness of the referring clinician; trust in the intentions for experimentation; and reassurance in the safety and ethical conduct of clinical trials pertaining to the informed consent process and the ability to withdraw participation. Trustworthiness, however, is largely contingent upon whether the institutions conducting, funding, and/or supporting the research study hold credibility in the participants’ eye [30]. As a consequential component for clinical trial recruitment, especially when seeking diverse samples, this contingency requires efforts to remediate historical legacies of harm in research experienced by various marginalized communities in order to establish trustworthiness [31–33].

Given the recency in the emergence of COVID-19 clinical trials, there is limited literature assessing participant engagement and retention in COVID-19 research trials. Unique to the context of COVID-19 has been a surging use of teleconferencing platforms (e.g., Zoom) to conduct research, transcending geographical constraints to increase participant recruitment for larger sample sizes [34]. Yet, these shifts to virtual settings for clinical trial administration necessitates exploration into factors that facilitate or hinder participant engagement and retention. Seeking to address this gap, the present study examined participants’ motivations to enroll in remote COVID-19 trial of Azithromycin, a well-established antibiotic drug, for mild-to-moderate symptoms of COVID-19. Understanding key factors that influence decision-making surrounding COVID-19 trial participation offers valuable insights for improving recruitment and retention strategies, ultimately advancing our understanding of and responses to research mobilization during times of crisis.

## Methods

### Research design

This research utilized evaluative qualitative methods to longitudinally assess participants’ experiences with and perspectives of the remote study procedures developed in the ACTION study. The ACTION study, conducted in collaboration with the University of California, San Francisco and Stanford University, aimed to determine the effectiveness of Azithromycin in treating mild-to-moderate symptoms of SARS-CoV-2 infections among COVID-19-positive outpatients [13]. The longitudinal qualitative evaluation was conducted at two different timepoints approximately one year apart (timepoint 1: December 2020–March 2021; timepoint 2: January–February 2022). The purpose of our study was to gain insights into the acceptability and feasibility of the remote study procedures. Our qualitative evaluation did not explore the therapeutic properties or usage of Azithromycin. Instead, the study included interviews to explore the experiences and perspectives among participants who had previously been diagnosed with COVID-19 and had participated in the ACTION clinical trial.

### Participants and data collection procedures

Nineteen participants were interviewed at timepoint 1 (T1). After being contacted again for a follow-up interview, 11 of those participants were interviewed at timepoint 2 (T2). We applied a purposive sampling strategy [35] to recruit study participants who consented to being contacted for future research studies. The purposive sampling strategy facilitated the intentional recruitment of participants who could provide diverse insights of and experiences with COVID-19, including participants aged 65 and older, cisgender women, participants with multiple comorbidities, self-identifying members of marginalized racial and/or ethnic groups, and participants who also consent to the optional swab sub-study. We established the following inclusion criteria: (1) aged 18 and older; (2) received a positive COVID-19 test result; (3) resided in the US; and (4) obtained capacity to consent. Full methods and demographics of qualitative evaluation have also been published elsewhere [36].

Eligible participants were initially contacted via email. Upon expressing interest, they were given a written consent form to review and sign before the interview. A calendar invitation with Zoom access information and a Zoom instructions sheet were then provided to ensure smooth participation. The interviews themselves lasted between 20 and 50 min, and were audio recorded.

A semi-structured interview guide was created with collaborative efforts from the ACTION study team and consisted of the following domains: experiences in a remote clinical trial during the COVID-19 pandemic, forms of social support, and access to healthcare. Ethics approval was obtained for all study procedures from the University of California, San Francisco (IRB protocol #20-32453) (IRB protocol #20-32453) and the University of Toronto (REB protocol #23729). Monetary incentives were not provided to participants enrolled in parent trial. As such, there was no economic renumeration for participation in the qualitative evaluation and was based on a voluntary basis.

### Data analysis

Following the conclusion of each timepoint, audio-recordings were de-identified, transcribed verbatim, and analyzed using immersion crystallization; an inductive and deductive method of identifying parent themes and child themes within the coding process [37]. Preliminary themes identified from T1 interviews were independently assessed and collaboratively refined; codified parent and child themes were then outlined in a structured codebook. All coding was conducted in Dedoose Version 9.0.17, a web application for managing, analyzing, and presenting qualitative and mixed method research data, (2021); Los Angeles, CA, USA: SocioCultural Research Consultants, LLC. Two research team members double-coded 25% of all T1 transcripts to ensure a consistent approach [38], and any coding differences encountered were discussed and reconciled at regular team meetings [39]. Coding discrepancies and resolutions were documented within an online audit trail and shared across research team members for timely and accessible information dissemination [40, 41]. Subsequently, this process was replicated following the completion of T2 interviews to conclude data analysis.

## Results

A total of 30 interviews were conducted at two timepoints: nineteen interviews (*n* = 19) at timepoint 1 (T1) and eleven interviews (*n* = 11) at timepoint 2 (T2). The participants had a mean age of 47 years old at the first timepoint, were predominantly white (75%), were women (63%), reported no co-morbidities (74%), and resided mostly in California (58%).

Through the interviews, several key themes emerged regarding participants’ initial engagement, retention, and continued involvement in COVID-19 research: (1) motivations for trial participation; (2) factors facilitating participant engagement in COVID-19 research; (3) elements that encouraged retention in the trial; (4) perceived challenges associated with the trial; and (5) participants’ recommendations for future studies. Subsequently, we conducted an additional analysis of the data that took into account the participants’ social contexts, including their demographics. This analytical approach enabled the identification of unique factors associated with their backgrounds, particularly their privileged status, and facilitated the contextualization of the data within this social framework. Specifically, this approach shed light on the participants’ motivations to enroll in the clinical trial, further enriching our understanding of their engagement and retention in the study.

### Part 1: motivations for participation

#### Altruism

Participants commonly shared motivations for initial interest and engagement in COVID-19 science as it related to altruism. During T1 interviews, participants stated that the prevailing uncertainty and confusion surrounding COVID-19 prevention and treatment influenced their decision to participate in research, with the aim of “contribut[ing] to the greater good” (T1, 56 y/o, W, CA). Similarly, participants acknowledged that amidst the confusion, their involvement in studies granted them agency and power. As a 47-year-old woman from California expressed, “I signed up for every study I could because I believed I could make a valuable contribution as a data point.” Participants also shared recognition of the social implications associated with participating in research:

Never before would you have a study where people are scrambling to get in, you know, voluntarily, right? (…) I think people recognize that the faster they can get science caught up, we can get back to normal, right? So, in some ways, that’s good for them, right? (…) If something happens in the future, you should think about participating ‘cause it helps the greater good. (T1, 45 y/o, M, TX)

During T2, when participants were probed about their motivations for initial participation, many highlighted the pivotal role of volunteering to advance scientific knowledge. As elucidated by one participant, “I comprehend that studies heavily rely on the voluntary commitment of individuals who are willing to dedicate a portion of their time to propel scientific progress… I strive to ensure that others can also benefit from the eventual findings and conclusions derived from this study; hence, that serves as a motivating factor for me” (T2, 37 y/o, M, CA).

#### Perception of potential benefits

Participants also cited perceived self-benefits of engaging in COVID-19 research as central to their decision to participate. Especially since initial participation occurred when COVID-19 vaccines and treatments were still under development, the possibility of being assigned a pharmaceutical treatment through a clinical trial was described by one participant as holding the potential to “[help them] heal faster [from COVID-19] or somehow [making them] less risky to [their] parents (…) who are in their 80s” (T1, 47 y/o, W, CA).

Another participant, a 45-year-old man from Texas, highlighted the importance of data sharing, stating, “I am in favor of sharing data; the more data we have, the better. If nobody participates, progress will never be achieved.” This participant further emphasized the connection between supporting COVID-19 science and the desire to return to normalcy.

### Part 2: facilitators for engagement in COVID-19 research

#### Social network: friends, family and social media

Initiation and engagement in COVID-19 research were commonly facilitated through social networks, such as friends and family, as well as social media. Participants at both timepoints expressed that they learned about the study through their family members, who sent them the study link, and commented on the importance of spreading the word about these studies: “It’s funny how people just don’t know, or think about [these studies], so the word has to be spread as much as possible” (T1, 50 y/o, W, CA).

The power of word-of-mouth not only disseminated information about the study but also encouraged participation. As one participant shared, “A friend of mine [that] I used to live with [who was] not involved in the study (…) told me that they’re doing this study and that they’re looking for people, and that I should do it (…) So I did and my roommate participated as well” (T1, 35 y/o, M, CA). Other participants found out about the study through internet sources such as “on a Reddit group [where] there was a thread about different research studies regarding COVID” (T1, 54 y/o, W, MI), “on Facebook” (T1, 39 y/o, W, NY), and by “Googling for studies that were enrolling [participants for COVID-19 research]” (T1, 47 y/o, W, MO). Leveraging social media platforms proved to be an important resource for sharing information about COVID-19 among the participants.

#### Healthcare professionals

Participants also mentioned the role of primary care physicians in supporting their enrollment in the parent study. One participant shared how “[her] primary care doctor gave [her] name to UCSF” (T2, 74 y/o, W, CA). Other participants became aware of this study through their healthcare centre: “My medical information was accessible because [I receive care at a medical institution where] researchers within their world are able to access people with certain conditions and then ask you to volunteer for participation” (T1, 71 y/o, M, CA).

#### Trust in scientific process

During T1 interviews, participants expressed satisfaction and affirmed their decision to participate in the trial, despite Azithromycin proving ineffective for treating mild-to-moderate symptoms of COVID-19. When asked about the impact of these findings on their decision to participate, many participants emphasized their contentment and expressed no regrets. One participant aptly articulated their perspective, stating that they did “not hav[ing] any regrets” about participating: “That drug [Azithromycin] didn’t come out as like something that [worked], so you tick that off, and then maybe the next one is something that is good – that’s how science works” (T2, 47 y/o, W, MO).

In parallel, other participants shared similar sentiments, emphasizing the value of null findings in research. They recognized that even when treatments did not yield the desired results, their contributions to scientific knowledge enabled the removal of a potential option, narrowing down the possibilities: “Not having it work could be just as valuable. Now, it’s one less variable [and] one less path to go down” (T2, 45 y/o, M, TX). One participant mentioned that these outcomes were “not gonna stop [them] from participating in another trial if one comes up that [they] fit in” and narrated involvement in other COVID-19 research studies: “When I had finally recovered [from COVID-19], I went down and I donated my plasma” (T2, 39 y/o, W, NY). Despite some disappointment in outcomes, one participant described trust in the scientific process, content in their participation, and low study burden as a motivator for future participation in COVID-19 research studies:

Listen, this is how I looked at it. […] Were the results what I wanted? No. But, I mean, come on, this is science. You make a hypothesis, you run the test and you figure out the answer, and it may not be what you want, but at least you know, so you can move on and do something else. So 100% I was happy to participate. […] If I could help out in another way – and it was like not a difficult thing to participate in, like it was easy to do, not a problem (T2, 49 y/o, W, GA).

These accounts demonstrate participants’ informed perspectives, resilience, and trust in the scientific enterprise, underscoring their commitment to contributing to advancements in COVID-19 research despite potential setbacks. This perceived benefit in access to medical care was relevant for both treatment and having an increased access to medical knowledge, as described:

I’ve participated in [four studies] (…) and the motivation is [that] I’m retired, the participations are easy, and there’s a benefit to participating in medical studies because you may find out more about your medical condition than you would otherwise have found out. So, that has a personal value. You might find something that should be treated, and timely treatment could be helpful. (T1, 71 y/o, M, 71, CA)

Other participants described a shared sentiment, grounded in confidence of the scientific process, that underscored decisions to engage in and support COVID-19 research. For example, “I trust science [and] science is the only way we’re gonna get through [the COVID-19 pandemic]” (T1, 43 y/o, M, CA). Willingness to participate in COVID-19 research was also bolstered by name recognition, due to the affiliated Universities and funder’s reputation, as well as participants’ familiarity with the study drug, Azithromycin: “It was just a random antibiotic, Azithromycin, and then also, [the University of California, San Francisco (UCSF)] (…) So, I felt comfortable” (T1, 51 y/o, W, CA). Participants’ prior experiences receiving healthcare through UCSF, where “[healthcare providers have] done nothing but good stuff”, further contributed to participants’ trust in the study (T1, 42 y/o, M, CA).

### Part 3: factors facilitating participant retention

#### Clear instructions

Once enrolled, participants found the study procedures to be straightforward, which facilitated their continued engagement. Participants explained that study procedures were “easy to do [from] the initial steps” and, therefore, “didn’t impact [their] life at all” (T1, 39 y/o, W, NY). In particular, participants spoke about the “simple directions [that were] easy to follow”, making this “a really easy project” to participate in (T1, 51 y/o, W, CA), including the intake “survey [that was] short and to the point” (T1, 39 y/o, W, NY). For example, many participants noted the helpful “diagrams and explanations [provided for collecting study] samples” (T1, 45 y/o, M, NM) and “clear instructions [with good] follow-up” from the study team (T1, 54 y/o, W, MI). Majority of participants described their participation in the trial as “really straightforward” (T1, 45 y/o, M, NM), due to clear study instructions, expedient delivery of study materials, and clear communication with the study team.

#### Willingness to contribute with low effort

Within the qualitative evaluation study, participants expressed their perception of low burden and minimal commitment as influential factors for their involvement in the study. One participant described the experience as simply “talking [to the interviewer], and [the interviewer] writing and hearing [them]. [The commitment is] nothing. […] Yet, it could help so much […]. That’s how people learn” and further stated: “I’m totally willing to be a ‘guinea pig’, up to a certain extent” (T1, 50 y/o, W, CA).

Similarly, another participant shared their perspective, stating, “I figured, anything I’ve got, you can have it. You want me, you want me to swab my nose? Okay. Or my mouth, or my gums, or whatever? That’s fine. I think a lot of times, there’s enough people who are afraid of what will happen to their data, or their information, or whatever. It stops them from participating. […] I’m happy to be part of that [research] (T1, 54 y/o, W, MI).

### Part 4: perceived challenges

#### Challenging instructions

While support from social networks and ease of participation were key components for engagement and retention in COVID-19 research, participants also reported some challenges that impacted complete engagement. Although participants generally found the study instructions to be clear and straightforward, some individuals who had prior experience with the study materials expressed concerns about those who lacked familiarity being able to use them correctly. One participant explained:

A part of the kit is that you have to secure the samples with Parafilm, and they don’t really tell you how to use the Parafilm. I happen to have a prior degree in chemistry, so I know how to use Parafilm, but I can imagine if you’ve never seen this stuff before… it could be a little bit unusual to use. (T1, 32 y/o, M, CA)

Similar concerns were raised by a few participants who set personal boundaries when agreeing to provide biological data in the swab study, which was an optional component of the study. For instance, one participant shared their comfort levels and choices regarding the types of biological samples they were willing to provide, stating: “I did skip the anal part, though. That was not for me. […] I was good with nose and mouth” (T1, 47 y/o, W, CA).

#### Perceived risks

It is important to note that many participants interviewed at T1 shared that they may not be willing to engage in a novel treatment trial due to unknown risks. Many expressed “apprehensions” and concerns for safety if the intervention was “a drug that [they] didn’t know about or [they] couldn’t research” (T1, 51 y/o, W, CA). Some participants, who described Azithromycin as a “common drug”, weighed their experienced risks of COVID-19 infection against the unknown benefit of a novel treatment: “I didn’t feel like [COVID-19] would impact my life to an extent where I would wanna try any sort of experimental drug without knowing the side effects” (T1, 35 y/o, M, CA).

### Part 5: recommendation for future research

#### Participants suggestion for more advertisement

Although informational supports received from family, friends, and networks facilitated initial interest in COVID-19 research for participants engaged in this trial, participants recommended “more advertising” of the study by the research team (T2, 51 y/o, W, CA). Questioning “if the average person knows that there are clinical studies that can be done in [order] to help other people”, participants stated that their only suggestion was “to let people know that there are studies out there (…) [and] amplifying the voice that this [research] is going on” (T1, 51 y/o, W, CA).

#### The importance of sharing study results

Among participants interviewed during T1, few expressed a strong interest in receiving the results of their participation in the study. One participant explained: “I’d like to know (…) the study results, [they] would be helpful to see” (T2, 71 y/o, M, CA). For some participants, having access to study results would be a way of knowing “how many people [the study drug] actually helped” and “the end result of the study” (T1, 42 y/o, M, CA). Other participants stated that results would be helpful to “discuss it with [their] physician”, although clarifying that they “don’t need to have the physician be a filter of the data” (T1, 71 y/o, M, CA). Similarly, over 20% of participants requested to be emailed articles published from this qualitative evaluation study Table 1.

**Table 1:** Participant demographic characteristics

Characteristic	*n* (%)
Mean Age	47
Women	19 (63%)
Race/Ethnicity	
Afro American	1 (5.3%)
Afro Caribbean	1 (5.3%)
Asian American	2 (10.5%)
Black	1 (5.3%)
East Asian	2 (10.5%)
Hispanic	3 (15.8%)
Indian American	2 (10.5%)
South Asian	2 (10.5%)
Southeast Asian	2 (10.5%)
White	15 (75.0%)
Co-morbidities	
None	14 (73.7%)
Asthma	2 (10.5%)
Chronic Kidney Disease	1 (5.3%)
Diabetes	1 (5.3%)
History of Cancer	1 (5.3%)
Hypertension	2 (10.5%)
State	
California	11 (57.9%)
Georgia	1 (5.3%)
Michigan	1 (5.3%)
Missouri	1 (5.3%)
New Mexico	1 (5.3%)
New York	3 (15.8%)
Texas	1 (5.3%)

Based on: Perez-Brumer et al. [36]

## Discussion

These findings underscore the utility of embedded qualitative methods within clinical trials and how they can contribute to a broader understanding of the factors influencing participation, particularly in COVID-19 clinical trials. Further, it contributes valuable insights for enhancing recruitment and retention strategies that extend beyond COVID-19 clinical trial administration, shedding light on the nuances of motivations to participate in a remote, COVID-19 clinical trial during unprecedented times. Our findings revealed several key themes related to participant engagement and retention in COVID-19 research. The motivations for trial participation centered around altruism and the perception of potential benefits. Participants expressed a strong desire to contribute to the greater good and recognized the importance of their voluntary commitment to advancing scientific progress. Further possible benefits included potential access to the pharmaceutical intervention, contingent upon whether it was deemed effective, for quicker healing or reduced risk to vulnerable family members, paralleling previous literature [42]. The facilitators for engagement in COVID-19 research included social networks, healthcare professionals, and social media. Participants often learned about the study through friends, family, or online platforms, highlighting the power of word-of-mouth and the importance of spreading information about research studies.

Factors that helped retain participants in the study included clear instructions and the perception of low effort and burden. Participants found the study procedures to be straightforward and easy to follow, which facilitated their continued engagement. The willingness to contribute with minimal effort and the desire to be part of the research process were strong motivators for participants. These findings are consistent with those in the current literature, highlighting logistical support (i.e., where, when, and how to participate) as a key facilitator for clinical trial engagement, seeking to reduce participant burden and therefore increase willingness to participate [43, 44]. Yet, it is also important to note that participants in this study expressed a sense of pride and satisfaction in being able to contribute to scientific knowledge and progress.

Participants exhibited a high level of COVID-19 health literacy and actively sought information to participate in the study, blurring the lines between expert and lay knowledge. This recognition raises important considerations about enhancing the accessibility of research information, necessitating the expertise of participants in the design and implementation of future studies while also considering flexibility in research methodologies needed to recruit and retain participants [45]. For instance, research conducted by Perrins et al. found success with retaining of culturally and linguistically diverse participants during the COVID-19 pandemic by adjusting research methodologies, ultimately maintaining study power [46]. The findings from our study also emphasize the informed perspectives, resilience, and trust that participants had in the scientific enterprise, highlighting their commitment to contributing to COVID-19 research despite potential setbacks. Broad skepticism of clinical trials and pharmaceutical interventions, especially among members of marginalized communities, are rarely attributed to an individual clinician or researcher but rather the overall systems of health care [47]. One example observed during the COVID-19 pandemic involved the tensions between mass vaccination efforts to achieve herd immunity and circulating mistrust of COVID-19 vaccines [33]. Yet, our findings show that trust in the scientific research process, academic and funding institutions, encouragement from healthcare professionals (e.g., primary care physicians), and familiarity with Azithromycin played significant roles in participants’ decisions to participate in the study. These findings also reinforce the significance of fostering trust to promote participation in clinical trials, while also emphasizing need for diverse representation and inclusivity in research endeavors.

Our findings align with prior research that underscores the importance of trust in healthcare, health systems, media, politicians, and the state in influencing trial participation [48]. Conversely, a broad lack of trust in pharmaceutical companies, physicians, hospitals, and researchers has been associated with negative attitudes towards participating in clinical trials.

Abdelhafiz et al. conducted a multinational study examining the knowledge, attitudes, and perceptions of individuals in Egypt, Saudi Arabia, and Jordan regarding participation in COVID-19 clinical trials [49]. They found a significant correlation between a lack of trust in pharmaceutical companies, physicians and hospitals, and scientists, and negative attitudes towards participating in COVID-19 clinical trials. In parallel with their findings, our study revealed that trust in the institution, funders, and the study drug influenced participants’ decision to participate. Wentzell and Racila also demonstrated that trust in science and its institutions can serve as motivating factors for trial participation [50]. Participants’ pre-existing knowledge of trial processes contributed to their perception of the trial as “safe.” Similarly, participants in our study were motivated by their desire to contribute to scientific progress. Ridde et al. further confirms the vital role of trust in COVID-19 trial participation, encompassing not only trust in healthcare and health systems but also trust in media, politicians, and the state [48].

It is important to note that participants’ engagement in clinical trials can be linked to social characteristics leading to variations in participation. For instance, Black participants exhibited high COVID-19 vaccine trial rejection compared to Arab, Chaldean, Middle Eastern, North African (MENA) groups and Hispanic groups [51]. When considering educational background, groups with a graduate or college level of education were more likely to participate in trials compared to those with a high school level of education. Our participants exhibited a high level of COVID-19 related health literacy and actively sought information to determine whether to participate. Of note, our sample primarily consisted of White upper-class women, which is consistent with the findings from Abdelhafiz et al. who found that three-quarters of their participants had prior knowledge of clinical trials, often associated with higher education, with most respondents holding at least a university degree [49].

However, there were also challenges and perceived risks that affected participant retention. Some participants raised concerns about challenging instructions, particularly for using certain study materials. Clear and explicit instructions were important for ensuring participants’ understanding and ability to correctly perform study procedures. Additionally, participants expressed apprehensions about unknown risks associated with novel treatment trials [52, 53]. Overall, this study contributes valuable insights into the motivations and factors influencing participant engagement and retention in COVID-19 research. Moreover, as a remote study, it presents a unique opportunity to ease enrollment and continuity in clinical trials, as documented previously in the literature [52], to alleviate logistical barriers, albeit isolated to those who obtain access to and literacy of technology. Understanding these factors can inform the development of effective recruitment and retention strategies for future studies and diseases. Considering the increasing number of studies adopting a remote approach, higher recruitment rates, improved compliance, lower drop-out rates, and faster completion seen in clinical trials may be advantageous to traditional clinical trials, when feasible [54]. Future studies should aim to include more diverse populations and investigate additional factors that may influence participation in COVID-19 trials, such as trust, information accessibility, and logistical barriers. By addressing these factors, we can enhance the recruitment and retention of participants and further advance our understanding and response to the global health crisis posed by COVID-19.

### Limitations

It is crucial to acknowledge the limitations of this study to provide a comprehensive understanding of its scope and implications. The sample size of the study was relatively small and did not include people who chose not to participate nor those who chose not to be contacted for future research and whose views on trial participation may be less favorable. Future research endeavors should aim to include a larger and more diverse sample, encompassing individuals from various demographic backgrounds, ethnicities, and geographic locations. By doing so, a more representative and inclusive dataset can be obtained, allowing for a broader range of perspectives and experiences to be considered.

Furthermore, it is essential to consider the social context within which this research took place. The clinical trial investigated a drug that had already been approved and deemed safe for use. Unlike experimental drugs with unknown risks, the tested drug had a known safety profile, providing an important distinction in terms of participant safety. It is crucial to acknowledge that studies involving experimental drugs may entail additional risks and uncertainties, warranting further ethical considerations and precautions.

## Conclusion

Our findings reveal that barriers and facilitators to participation in COVID-19 trials are closely linked to participants’ social characteristics, such as their professional background, extensive knowledge, and active engagement in seeking information about the trial process. Trust in the research process and the involved institutions also emerged as a crucial factor to both engage and stay engaged in research. It is important to note that future studies should encompass participants with diverse demographic characteristics to more meaningfully engage, design tailored recruitment and retention strategies for more diverse research participants. This study highlights the importance of incorporating embedded qualitative methods within clinical trials by illustrating valuable insights based on lived-experience participating in COVID-19 research to strengthen recruitment and retention strategies, and enhance informed participation in future clinical trials.

## Data Availability

To inquire about access to original transcripts, please email the corresponding author (Amaya Perez-Brumer: [a.perezbrumer@utoronto.ca](mailto: a.perezbrumer@utoronto.ca) )

## Data availability

To inquire about access to original transcripts, please email the corresponding author (Amaya Perez-Brumer: a.perezbrumer@utoronto.ca).
